# 

**Published:** 1906-05-26

**Authors:** 


CONTENTS.

**Chemists and Companies** - - - - - - - 131
**Hospital Dinners and Annual Meetings** - - - - 132
**Annotations** - - - - - - - - - - - 133
**Current Professional Opinion** - - - - - - 134
**Hospital Clinics—**
The Treatment of Shock and Collapse following Surgical Operations - - - - - - - - - - 135
Broncho-Pneumonia - - - - - - - - - 138
Malignant Endocarditis treated by Antistreptococcic Serum - 140
New Remedies and Results - - - - - - - - - - 141
The Society for the Study of Diseases in Children - - - 141
**Medical Appointments and Vacancies** - - - - 142
**The Book World of Medicine and Science** - - - 143
**New Appliances and Things Medical** - - - - 143
**Hospital Administration—**
Motor-cars for Doctors - - - - - - - - - - 144
Current Hospital Topics - - - - - - - - - - 145
Mr. P. J. Michelli - - - - - - - - - - - 146
Hospital Meetings - - - - - - - - - - - - 147

*NURSING SECTION.*

**Notes on News from the Nursing World—** Matron Applicants for Office—An English Nurse and the San Francisco Disaster—An Institutional Badge—Fresh Friction at Exeter Infirmary—The Exchange Principle—A Patriotic Nurse—An Under-staffed Poor-law Infirmary—Cash instead of Uniform—Vacancies in the Army Service—Imperial Military Nursing Service—St. George's Hospital Nurses and the Pension Fund—The Ladies' Association of Guy's Hospital—Westminster Hospital Ladies' Association—The Mothers' Union and Nurses—Promotions at Camberwell Infirmary—New Nurses' Club—Missionary League—The Nurses of Holborn Union Infirmary—For the East London Nursing Society—Short Items - - - - - - - - - - - 113-116
**The Nursing Outlook** - - - - - - - - - 117
**Abdominal Surgery** - - - - - - - - - - 118
**The Nurse's Clinic** - - - - - - - - - 119
**Training in Massage** - - - - - - - - - 119
**The Earthquake in San Francisco** - - - - - 120
**The Nurses of Salisbury Infirmary** - - - - - 122
**Presentations** - - - - - - - - - - - 123
**The Training and Supply of Midwives** - - - - 123
**Sarah Gamp v. the Modern Nurse** - - - - - 124
**The Nurses' Stalls at the King's College Hospital Fete** - - - - - - - - - - - - - 124
**Everybody's Opinion** - - - - - - - - - 124
**Appointments** - - - - - - - - - - - - 125
**Novelties for Nurses** - - - - - - - - - 125
**Notes and Queries** - - - - - - - - - - 126
**For Reading to the Sick** - - - - - - - - 126

